# Effects of Combined Transcriptome and Metabolome Analysis Training on Athletic Performance of 2-Year-Old Trot-Type Yili Horses

**DOI:** 10.3390/genes16020197

**Published:** 2025-02-04

**Authors:** Liping Yang, Pengcheng Li, Xinxin Huang, Chuankun Wang, Yaqi Zeng, Jianwen Wang, Xinkui Yao, Jun Meng

**Affiliations:** 1 College of Animal Science, Xinjiang Agricultural University, Urumqi 830052, China; yanglp98@126.com (L.Y.); n37672846@126.com (P.L.); huangxx_97@163.com (X.H.); wck94214@163.com (C.W.); xjauzengyaqi@163.com (Y.Z.); wjw1262022@126.com (J.W.); yxk61@126.com (X.Y.); 2 Xinjiang Key Laboratory of Equine Breeding and Exercise Physiology, Xinjiang Agricultural University, Urumqi 830052, China; 3 Horse Industry Research Institute, Xinjiang Agricultural University, Urumqi 830052, China

**Keywords:** transcriptomics, metabolomics, trot, Yili horses, athletic performance

## Abstract

Objectives: Training is essential for enhancing equine athletic performance, but the genetic mechanisms that regulate athletic performance are unknown. Therefore, this paper aims to identify candidate genes and metabolic pathways for the effects of training on equine athletic performance through multi-omics analyses. Methods: The experiment selected 12 untrained trot-type Yili horses, which underwent a 12-week professional training program. Blood samples were collected at rest before training (BT) and after training (AT). Based on their race performance, whole blood and serum samples from 4 horses were chosen for transcriptomic and metabolomic analyses. Results: The race performance of the horses is dramatically improved in the AT period compared to the BT (*p* < 0.01) period. The transcriptome analysis identified a total of 57 differentially expressed genes, which were significantly enriched in pathways related to circadian entrainment, steroid hormone biosynthesis, chemokine signaling, and cholinergic synapses (*p* < 0.05). Additionally, metabolomic analysis revealed 121 differentially identified metabolites, primarily enriched in metabolic pathways such as histidine metabolism, purine metabolism, and the PI3K-Akt signaling pathway. The integration of transcriptomic and metabolomic analyses uncovered five shared pathways, and further combined pathway analyses identified eight differentially expressed genes that correlate with 19 differentially identified metabolites. Conclusions: The current findings will contribute to establishing a theoretical framework for investigating the molecular mechanisms of genes associated with the impact of training on equine athletic performance. Additionally, these results will serve as a foundation for enhancing the athletic capabilities of trot-type Yili horses.

## 1. Introduction

The growth and transformation of the modern equine industry have resulted in an increased variety and number of horse events, as well as a heightened demand for sport horses [1]. Athletic training and husbandry management are the primary factors influencing the event results and overall performance in horses [2]. The phenotypic and physiological changes resulting from exercise training have been extensively studied. Adaptations within the organism, driven by exercise-induced alterations in muscle loading, energy demand, and calcium fluxes, can facilitate the timely detection of abnormalities, yielding beneficial effects on cardiorespiratory, endocrine, and neurological health [3]. There is notable variability in the internal environmental changes that occur in response to different training intensities. Endurance training enhances the body’s aerobic capacity and promotes a transition from carbohydrate to fat metabolism. Resistance training fosters protein synthesis and facilitates a slow-to-fast muscle transition rate. Additionally, neuromuscular training serves as a strategy to manage motor control, improve the motor–sensory system, increase dynamic joint stability, and reduce the risk of injury [4]. Research has demonstrated that exercise training can regulate glucose and lipid metabolism, improve insulin sensitivity and anti-inflammatory capacity, reduce oxidative stress, stimulate muscle protein synthesis and satellite cell activation, enhance muscle fiber size, decrease body fat, and increase muscle strength, thereby improving competitive performance [5]. Kowalik et al. found that plasma concentrations of muscle growth inhibitors increased in 20 Arabian horses after 8 months of endurance training [6]. Fernandez et al. found that tennis players who participated in five weeks of neuromuscular training exhibited significantly improved sprint speed and movement sensitivity during the later stages of training compared to the earlier stages [7].

Transcriptomics is a powerful tool for identifying gene expression signatures [8]. Metabolomics serves as a valuable method for identifying metabolites and elucidating changes in metabolite levels within biological systems under varying conditions [9]. Concurrently, high-throughput sequencing technology has been employed to identify genes with significant phenotypic effects, enabling the development of training programs tailored to an animal’s susceptibility [10,11]. Studies indicate that repetitive exercise training induces the emergence of new gene expression in resting muscles, likely reflecting the organism’s capacity to adapt to training and influence exercise-related phenotypes [12,13]. Genes relevant to specific biological contexts can be identified by comparing gene expression across different states, such as before and after a particular exercise, or among various tissues [14], including blood versus muscle [15], as well as across different breeds and hybrids [16]. Hou et al. observed significant reductions in the levels of alanine, aspartate, glutamate, and pantothenic acid, along with the down-regulation of genes such as *ENTPD3*, *ENTPD31*, and *CMPK2*, following 8 weeks of countercurrent swim training in zebrafish [17]. This analysis was conducted using a combination of transcriptomics and metabolomics. In a related study, Isung et al. found that high-intensity exercise leads to an increase in glutamate levels in skeletal muscle, which subsequently promotes the release of alanine to facilitate ammonia metabolism [18].

Training plays a vital role in enhancing overall musculoskeletal health and athletic performance in sport horses [19]. Previous studies have employed transcriptomics to investigate the impact of training on equine athletic performance [20]; however, relying on a single technique offers a limited perspective and fails to provide a comprehensive understanding of how training influences athletic performance. Joint multi-omics analysis serves as an integrative approach that combines various data types to illuminate the dynamics of an organism’s internal environment from multiple angles [21]. Yili horses, recognized as the first breed independently developed in China, exhibit notable traits such as exceptional adaptability and disease resistance. Consequently, this study focuses on trot-type Yili horses, implementing a 12-week specialized training program. We investigated the impact of training on the horses’ athletic performance at the molecular level using transcriptomic and metabolomic technologies, ultimately identifying new genetic markers associated with athletic performance. This research aims to provide a theoretical foundation for the development of more professional and effective conditioning and training programs.

## 2. Materials and Methods

### 2.1. Test Animals and Training Programmes

The trial involved 12 two-year-old trot-type Yili horses from Zhaosu Horses Farm in Xinjiang, which underwent a 12-week training program (Table 1). Whole blood and serum samples were collected from all 12 horses under resting conditions during the before-training and after-training periods. Weekly test races were organized, and the four horses that consistently ranked in the top four positions across all races were selected based on their athletic performance. Transcriptomic and metabolomic sequencing was subsequently performed on the whole blood and serum samples from these four horses.

### 2.2. Sample Collection

Use the Finish Timing System to record the horse’s race results. The blood of horses at rest during the BT and AT periods was collected and rapidly placed in liquid nitrogen, and subsequently placed in a −80 °C refrigerator for cryopreservation.

### 2.3. Transcriptome Analysis

#### 2.3.1. RNA Isolation, Library Preparation and Sequencing

Total RNA was extracted from the blood of horses in both the BT and AT groups using the TRIzol extraction kit (Thermo Fisher Scientific, Waltham, MA, USA), with four biological replicates included for each group. Pairwise end sequencing was performed on the Illumina NovaSeq 6000 (Illumina, San Diego, CA, USA), which involved the removal of reads with junctions, reads containing unidentifiable base information, and low-quality reads to ensure the acquisition of high-quality clean reads. The values of clean reads were compared against a known horse reference genome (https://ftp.ncbi.nlm.nih.gov/genomes/all/GCF/002/863/925/GCF_002863925.1_EquCab3.0/, accessed on 14 June 2024) to obtain information regarding the positioning of reads on the horse reference genome.

#### 2.3.2. Differentially Expressed Genes Analysis

Differentially expressed genes (DEGs) were identified among the various control groups using the DESeq2 package in R (version 4.4.1), which is based on the negative binomial distribution. Genes were considered differentially expressed if they met the criteria of |log_2_(Fold Change)| ≥ 1 and a *p*-value < 0.05.

#### 2.3.3. Functional Enrichment Analysis

Gene ontology (GO) and Kyoto Encyclopedia of Genes and Genomes (KEGGs) pathway enrichment analyses were conducted utilizing the NovoMagic cloud platform (https://magic.novogene.com/customer/main#/loginNew, accessed on 28 August 2024). A significance threshold of *p* < 0.05 was employed to identify crucial functional pathways.

#### 2.3.4. Protein Network Interactions Analysis

Mapping of identified DEGs to the Search Tool for the Retrieval of Interacting Genes (STRINGs) database (http://string-db.org/, accessed on 12 September 2024). PPI networks were built using Cytoscape software (version 3.10.0) and core genes were identified using the MCC model in the CytoHubba plugin.

### 2.4. Metabolome Analysis

Metabolites were extracted from the plasma of horses in the BT and AT, with four biological replicates in each group. All samples were thawed at room temperature, 100 µL of thawed plasma samples was transferred to EP tubes with 400 µL of 80% methanol, and 1 mL of samples was lyophilized with 100 µL of 80% aqueous methanol; vortexed and shaken, and left to stand on an ice bath for 5 min, and then centrifuged at 15,000× *g* for 15 min at 4 °C. A certain amount of supernatant was diluted with mass spectrometry grade water to 53% methanol, centrifuged at 15,000× *g* and 4 °C for 15 min, and then the supernatant was collected and injected into LC-MS for analysis. The default criteria for differential metabolite screening were VIP > 1, *p*-value < 0.05, and FC ≥ 1.5 or FC ≤ 0.667.

### 2.5. Combined Transcriptome and Metabolome Analysis

A joint analysis of the transcriptome and metabolome revealed overlapping pathways between these two biological domains. Differentially expressed genes and metabolites were screened from the transcriptome and metabolome and subsequently analyzed together using the Pearson correlation analysis method. Visual heat maps and networks were generated to illustrate the findings.

### 2.6. Statistical Analysis

The results of the competition were analyzed using the one-way analysis of variance (ANOVA) method in SPSS 26.0 software and the results are expressed as the mean ± standard deviation. Differences were judged at *p* < 0.05 and *p* < 0.01.

## 3. Results

### 3.1. Changes in Race Performance of Horses at Different Stages of Training

The horses’ race times were highly significantly lower in the AT than in the BT (*p* < 0.01) periods (Table 2). The horses’ race times were significantly lower in the mid-training period than in the BT (*p* < 0.05) period (Table 2).

### 3.2. Transcriptome Results Analysis

#### 3.2.1. Transcriptome Quality Control Analysis

The raw data of the blood transcriptome of horses in the BT and AT groups were 192,181,348 and 167,025,522, respectively, and 46,730,816 and 73,255,360 high-quality and valid data were obtained after quality control filtering of the raw data (Table 3). The percentage of Q20 bases was above 96.49%, the rate of Q30 bases was above 92.77%, and the GC content ranged from 51.34% to 58.40% (Table 3). When clean reads were compared to the horse’s reference genome, the average comparison efficiency for the eight samples was 71.37% (Table 3). Due to the specificity of the blood samples, the sequencing data were largely compliant and met the requirements for subsequent analyses.

#### 3.2.2. Differential Genes Analysis

**Figure 1:**
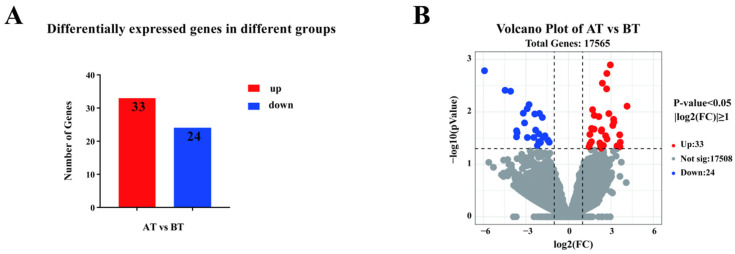
Transcriptome analysis of equine blood in AT versus BT. (**A**) The number of up- and down-regulated differentially expressed genes (DEGs). (**B**) Volcano plot for differential genes expression (*n* = 4).

A total of 57 DEGs, including 33 up-regulated and 24 down-regulated genes, were identified in the blood of horses in the AT versus BT groups (Figure 1A,B). *FOS*, *PRF1*, *CD3E*, *CCL5*, *HSD17B1*, and *TMPRSS6* were significantly up-regulated in the AT group compared to the BT group (*p* < 0.05), and *C1QTNF12*, *GATA1*, *CCR3*, and *ND5* were significantly down-regulated compared to the BT group (*p* < 0.05) (Table 4).

#### 3.2.3. Transcriptome Pathway Enrichment Analysis

**Figure 2:**
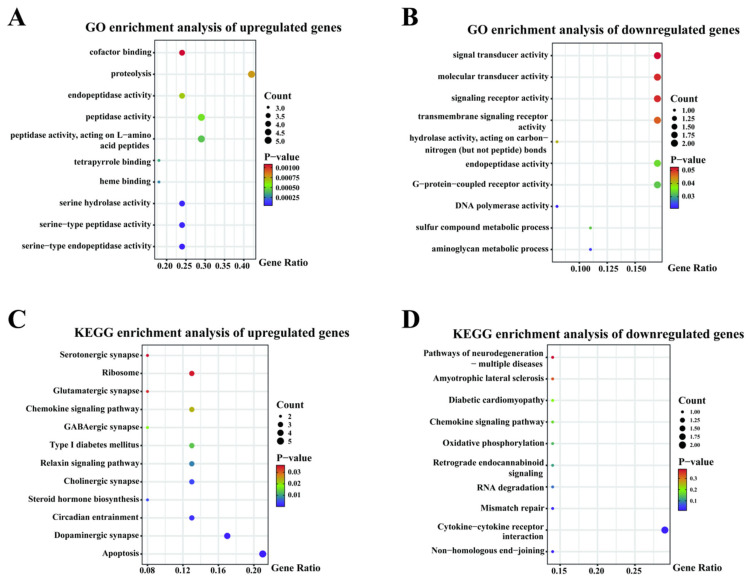
Analysis of differential genes enrichment in AT versus BT. (**A**) GO enrichment analysis of up-regulated genes. (**B**) GO enrichment analysis of down-regulated genes. (**C**) KEGG enrichment analysis of up-regulated genes. (**D**) KEGG enrichment analysis of down-regulated genes.

GO functional enrichment analyses of DEGs in the blood from horses in AT versus BT groups were analyzed, and the top 10 GO term entries in each GO category were selected for display. Up-regulated genes were enriched in 105 GO entries, of which they were significantly enriched in 34 GO entries (*p* < 0.05), mainly in GO entries related to G protein-coupled receptor binding, chemokine activity, heme binding, serine-type peptidase activity, and proteolysis (Figure 2A). Down-regulated genes were enriched in 50 GO entries, of which they were significantly enriched in 7 GO entries (*p* < 0.05), mainly in GO entries for the aminoglycan metabolic process, sulfur compound metabolic process, G-protein-coupled receptor activity, and DNA polymerase activity (Figure 2B). In KEGG pathway analysis, up-regulated genes were enriched in 101 pathways, of which they were significantly enriched in 24 pathways (*p* < 0.05). Twelve pathways were screened for the possible regulation of equine athletic performance, including apoptosis, the dopaminergic synapse, circadian entrainment, steroid hormone biosynthesis, the cholinergic synapse, the relaxin signaling pathway, type I diabetes mellitus, the GABAergic synapse, the hemokine signaling pathway, the glutamatergic synapse, ribosome, and the serotonergic synapse (Figure 2C). Down-regulated genes were enriched in 21 KEGG pathways, with significant enrichment in 3 KEGG pathways (*p* < 0.05). Screening was conducted of 10 pathways, including non-homologous end-joining, cytokine-cytokine receptor interaction, mismatch repair, RNA degradation, retrograde endocannabinoid signaling, oxidative phosphorylation, the chemokine signaling pathway, diabetic cardiomyopathy, amyotrophic lateral sclerosis, and the pathways of neurodegeneration-multiple diseases, which may modulate equine athletic performance (Figure 2D).

#### 3.2.4. Protein Network Interactions Results Analysis

**Figure 3:**
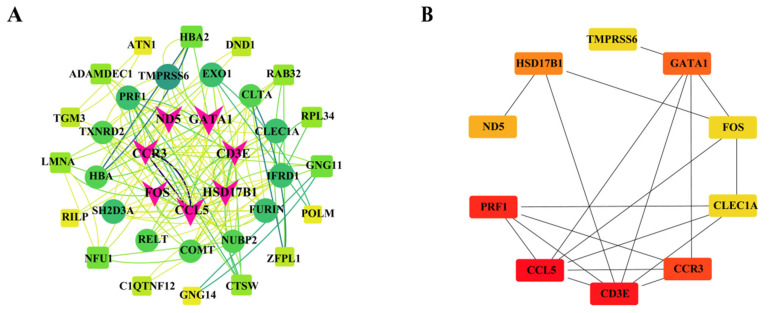
Interaction analysis of AT and BT differential genes protein networks. (**A**) Differential genes protein network interaction map. (**B**) Map of core genes significantly associated with the athletic performance of the trot-type Yili horses.

The protein-protein interaction (PPI) network was constructed by integrating differentially expressed genes (DEGs) with protein interaction data from the STRING database. Remove the disconnected nodes and keep the network with the most nodes (Figure 3A). A total of 10 core genes, including *CCL5*, *FOS*, *CD3E*, *HSD17B1*, *CCR3*, *GATA1*, *TMPRSS6*, *CLEC1A*, *PRF1*, and *ND5*, which may be significantly associated with the athletic performance of the trot-type Yili horses, were screened by the MCC model in Cytoscape software CytoHubba (Figure 3B).

### 3.3. Metabolome Results Analysis

#### 3.3.1. Metabolome Quality Control Analysis

**Figure 4:**
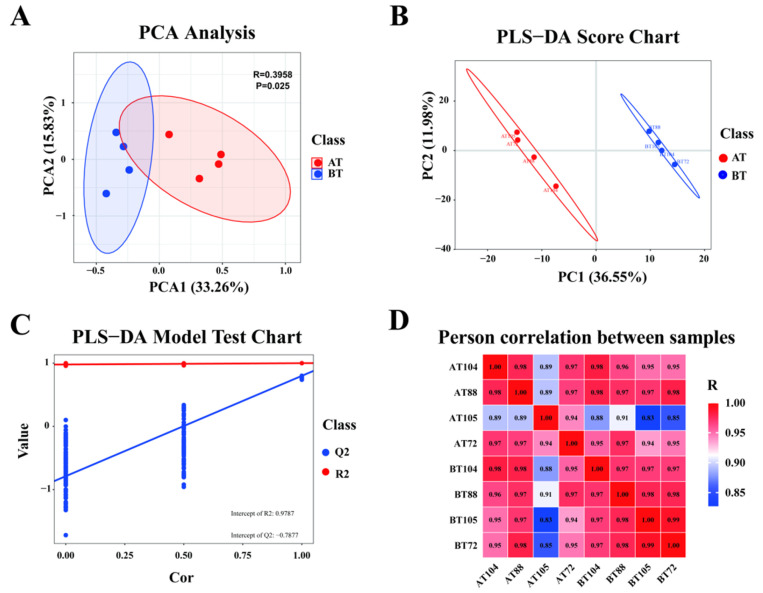
Quality control analysis of equine plasma in AT versus BT. (**A**) PCA score plot of the metabolome. (**B**) PLS-DA score plot of the metabolome. (**C**) PLS-DA model test of the metabolome. (**D**) Pearson correlation between samples.

To ensure the stability of the overall test, PCA and correlation analyses were performed on the test samples. PC1 and PC2 accounted for 33.26 percent and 15.83 percent of the total variation (Figure 4A). To better distinguish the differences between groups, PLS-DA analysis was performed on top of PCA analysis, which showed significant differences between groups (Figure 4B,C). The correlation between the samples tends to be closer to 1.00 (Figure 4D). The above results showed that there was a significant difference in the plasma of horses in the AT and in the BT groups.

#### 3.3.2. Differential Metabolites Analysis

**Figure 5:**
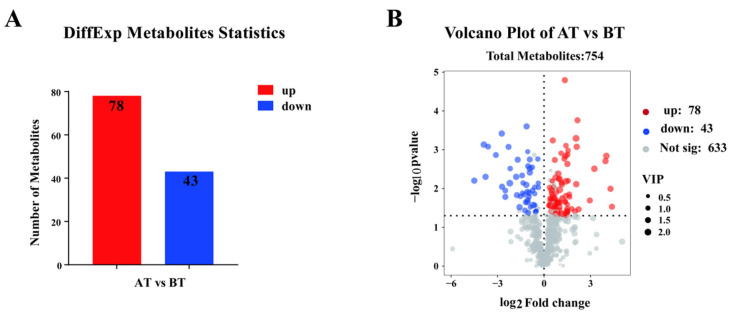
Metabolome analysis of equine plasma in AT versus BT. (**A**) The number of up- and down-regulated differentially expressed metabolites. (**B**) Volcano plot for differential metabolites expression (*n* = 4).

A total of 121 differential metabolites were screened in AT versus BT plasma, of which 78 differential metabolites were up-regulated and 43 differential metabolites were down-regulated in expression (Figure 5A,B). Differential metabolites that may be associated with equine athletic performance were screened, with dehydroepiandrosterone (DHEA), cis-aconitic acid, and carnosine all being significantly higher than in the BT group (*p* < 0.05), and pentadecanoic acid, asparagine, androsterone, and ergothioneine being significantly lower than in the BT group (*p* < 0.05) (Table 5).

#### 3.3.3. Metabolome Pathway Enrichment Analysis

**Figure 6:**
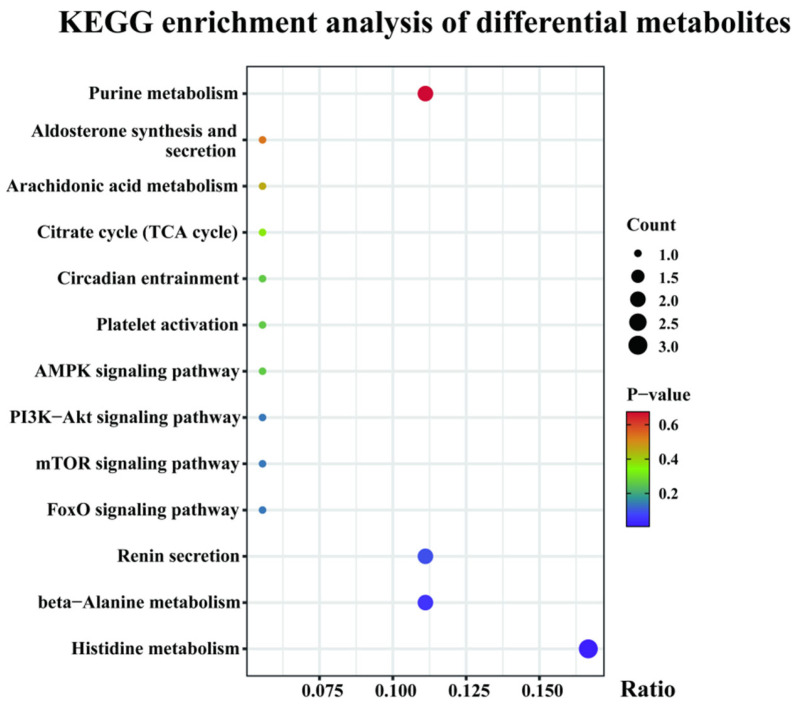
Analysis of differential metabolites enrichment in AT versus BT.

The differential metabolites were analyzed for KEGG functional annotation and pathway enrichment, as shown in this experiment. Differential metabolites were enriched in a total of 38 metabolic pathways, of which 3 differential metabolites were highly significantly enriched in the histidine metabolism (*p* < 0.01). Thirteen pathways potentially related to athletic performance were screened, including histidine metabolism, beta-alanine metabolism, renin secretion, the FoxO signaling pathway, the mTOR signaling pathway, the PI3K-Akt signaling pathway, the AMPK signaling pathway, platelet activation, circadian entrainment, the citrate cycle (TCA cycle), arachidonic acid metabolism, aldosterone synthesis and secretion, and purine metabolism (Figure 6).

### 3.4. Association Analysis Between Transcriptomic and Metabolomic Data

**Figure 7:**
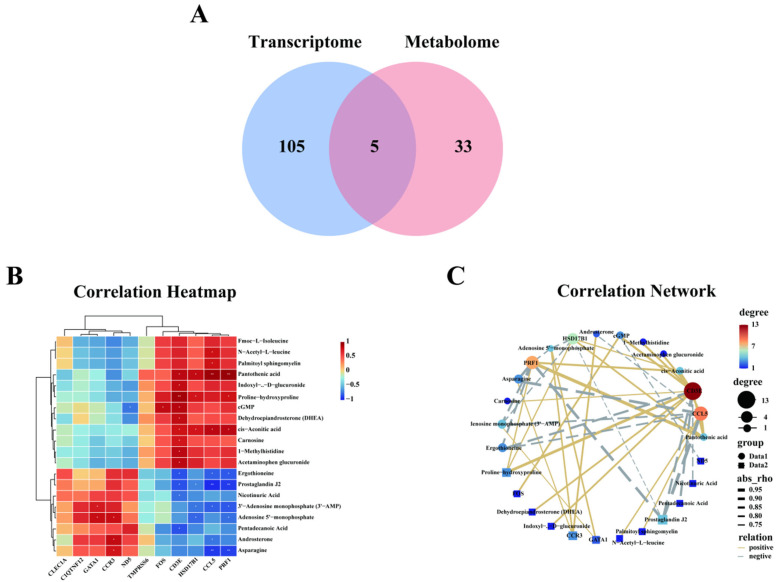
Integrated analysis of metabolomic and transcriptomic profiling of AT and BT. (**A**) Venn diagram of shared KEGG terms among transcriptome and metabolome. (**B**) Correlation heatmap of differentially expressed genes and metabolites for the integrated pathway. *, **: significant positive or negative correlation. (**C**) Correlation network of differentially expressed genes and metabolites for the integrated pathway.

To investigate the association between differential genes and differential metabolites in AT and BT blood, the KEGG enrichment pathway was integrated. The Wayne diagram results showed that there were five shared KEGG pathways for the differential genes and differential metabolites, namely circadian entrainment, the serotonergic synapse, the PI3K-Akt signaling pathway, the oxytocin signaling pathway, and the cAMP signaling pathway (Figure 7A). Correlation tests were conducted for 11 differentially expressed genes and 20 differential metabolites associated with athletic performance based on analyses of transcriptomic and metabolomic KEGG-enriched pathways. By Pearson correlation analysis of 11 differential genes and 20 differential metabolites, a total of 8 genes were significantly positively or negatively correlated with one or more of the 19 metabolites (Figure 7B,C).

## 4. Discussion

Regular long-term exercise training can induce a variety of adaptive responses in the body, which not only enhance glycolysis and fatty acid metabolism but also increase the sensitivity of the central nervous system and the pancreatic islet system, thereby strengthening the body’s exercise capacity [22]. Integrating transcriptomics and metabolomics provides a more precise understanding of the molecular regulatory mechanisms that condition athletic performance in trot-type Yili horses, thereby reflecting their physiological state.

Members of the FOS family are implicated not only in the pathogenesis of various diseases but also serve as reliable markers of neural activity and play crucial roles in the maintenance of skeletal cellular development [23]. In the present study, *FOS* was found to be up-regulated in AT, suggesting that *FOS* promotes the development of the horse’s brain and regulates the central nervous system. This up-regulation may lead to the increased excitability of the central nervous system, enhanced motor performance, and improved development of skeletal cells. Additionally, *CCL5* is known to regulate the movement of memory T lymphocytes, monocytes, macrophages, and eosinophils [24]. Related studies have indicated that *CCL5* is significant in promoting synaptic growth and memory formation, and it plays a role in central nervous system disorders, particularly those associated with neuroinflammatory processes [25]. In their study of related genes in mice, Szalay et al. found that *CCL5* up-regulated the expression of *IL-10* in both vascular smooth muscle cells and the brain. They demonstrated that *IL-10* promotes the differentiation of type 2 microglia and prevents the over-activation of pathological microglia, indicating a protective role for *CCL5* in the context of neuronal injury [26]. In the present study, *CCL5* expression was significantly up-regulated in AT, suggesting that *CCL5* activates eosinophil activity, maintains the body’s acid-base balance, and reduces the incidence of acid-base toxicity in horses. It is hypothesized that *CCL5* may play a role in regulating the homeostasis of the horse’s internal environment, thereby enhancing its athletic performance. Reißmann et al. sequenced the transcriptome of Kabardino horse blood following long-distance (70 km) and short-distance (15 km) endurance running. Their findings revealed that the pathways and genes associated with the activation of the inflammatory system, carbohydrate catabolic processes, lipid biosynthesis, NADP metabolic processes, as well as specific genes such as *ACOD1*, *CCL5*, *CD40LG*, *FOS*, and *IL1R2*, can be utilized to monitor equine exercise performance [27]. These results are consistent with those of previous studies.

Studies have shown that the PI3K-Akt, mTOR, and FoxO signaling pathways play central regulatory roles in carbohydrate, lipid, and protein metabolism by coordinately modulating biological processes such as apoptosis, energy metabolism, and oxidative stress [28]. Among these, mTOR integrates nutritional signals (e.g., amino acid availability), energy signals (ATP/AMP ratio), and growth factors (e.g., insulin/IGF-1) through its complexes (mTORC1/mTORC2) to regulate critical metabolic processes, including protein synthesis (e.g., via p70S6K activation), lipid metabolism, and mitochondrial biogenesis [29]. McGivney et al. identified through transcriptomic analysis that in thoroughbred horses exercised on a treadmill to maximum heart rate, mTOR signaling-related genes (*4EBP1*, *TSC2*, *VEGF*) were up-regulated in skeletal muscle 4 h post-exercise, demonstrating that exercise-induced stress enhances metabolic adaptation through the time-dependent modulation of the mTOR network [30]. Takegaki et al. further revealed that three sessions of resistance exercise in 18 male mice significantly activated the phosphorylation of the mTOR signaling marker p70S6K in skeletal muscle, confirming the cross-species conservation of the mTOR pathway function in exercise-mediated anabolism [31]. Our findings indicate that differentially expressed genes and metabolites were co-enriched in the PI3K-Akt signaling pathway, while differential metabolites were specifically enriched in the PI3K-Akt, mTOR, and FoxO signaling pathways. This suggests that during the experimental conditions, these pathways collectively regulate exercise performance outcomes by integrating growth factor signaling to modulate substrate metabolism, sensing amino acid and energy status for a dynamic balance of anabolism/catabolism, and activating lipolysis and antioxidant gene expression. *C1QTNF12*, a member of the C1QTNF family, plays a crucial role in glucose metabolism within the liver and adipose tissue by promoting glucose uptake in adipocytes and inhibiting de novo glucose production in hepatocytes through the PI3K-Akt signaling pathway. Additionally, *C1QTNF12* is involved in regulating inflammation, vascular remodeling, and cardiac fibrosis, which may contribute significantly to cardiovascular injury [32]. In the present study, the significant down-regulation of *C1QTNF12* in AT aligns with previous findings [33]. This suggests that *C1QTNF12* may mitigate inflammation in AT, enhance hormonal activity, regulate gluconeogenesis, and store substantial energy for the organism, ultimately leading to improved athletic performance.

Steroid hormones, classified as fat-soluble hormones, are primarily divided into two main groups: sex hormones and adrenocorticotropic hormones. These hormones are typically synthesized in various tissues, including the adrenal cortex, gonads (testes and ovaries), brain, placenta, and adipose tissue [34]. The adrenocorticotropic hormone, secreted by the adrenal cortex, plays a crucial role in regulating glucose metabolism. It not only inhibits sugar oxidation, which raises blood glucose levels, but also promotes the conversion of protein into sugar [35]. Additionally, it facilitates the retention of sodium ions while promoting the excretion of excess potassium ions, thereby regulating water and salt metabolism [36]. The secretion of sex hormones is controlled by gonadotropins originating from the pituitary gland. In the results of this study, the steroid hormone synthesis pathway emerged as significantly enriched in the transcriptome and metabolome concerning the effects of training on equine energy transport performance. Notable differential genes and metabolites, including *HSD17B1*, testosterone, and dehydroepiandrosterone, were enriched within this pathway. *HSD17B1* is biologically significant as it catalyzes the conversion of androstenedione to testosterone, facilitates the reduction of DHEA to androstenediol, and metabolizes dihydrotestosterone, the most potent androgen, into 3β-diol and 3α-diol [37]. In the present study, we observed that the expression of *HSD17B1* was up-regulated in AT, indicating that training enhances the in vivo function of the *HSD17B1*, which leads to the increased secretion of steroid hormones, including testosterone, in the organism. Testosterone is a crucial anabolic steroid hormone that plays a significant role in the growth and maintenance of skeletal muscle, enzyme proteins, bone, and red blood cells, and it also contributes to neurological functions [38]. Relevant studies have demonstrated that testosterone can directly influence androgen receptors on osteoblasts and osteoclasts, resulting in increased trabecular bone formation, the inhibition of osteoclast activity, reduced bone resorption, and concurrently enhanced muscle strength and bone mineral density. Additionally, it promotes oxidative muscle metabolism, thereby improving athletic performance [39]. Hackney et al. found that prolonged endurance exercise induces a biphasic response in testosterone levels, characterized by an immediate increase post-exercise followed by a subsequent decrease during the recovery phase [40]. Our findings revealed an increase in blood testosterone levels after training compared to before training levels; however, testosterone levels during the recovery phase were not assessed, indicating a need for further investigation. DHEA, primarily synthesized by the adrenal cortex and gonads [41], has several biological roles, including improving glucose tolerance, increasing insulin levels, exerting antidiabetic effects, enhancing endocrine system activity, lowering cortisol levels, restoring impaired immune responses, participating in the synthesis of various adrenal hormones, and enhancing T cell and B cell immune functions [42]. Relevant studies have demonstrated that DHEA reverses vascular remodeling, enhances vascular endothelial cell function, and reduces oxidative stress in the body [43]. In the present study, an increase in blood levels of DHEA was observed in horses after exercise compared to before exercise. This could be due to DHEA improving tolerance and enhancing athletic performance by increasing insulin levels and exerting anti-diabetic effects in the body [44].

Histidine is an essential amino acid for humans, mammals, fish, and poultry. As a functional amino acid, it exerts specific metabolic effects beyond its role in protein metabolism [45]. Histidine is involved in various metabolic pathways; it can be methylated to form 1-methyl or 3-methylhistidine, converted to imidazole-pyruvic acid by transaminases, condensed with β-alanine to produce carnosine and goitrogens, or decarboxylated to generate histamine [46]. In the present study, histidine metabolism was the pathway significantly enriched in the metabolomic analysis of the effects of training on equine energy transport performance, with differential metabolites such as myostatin, ergothioneine, and 1-methylhistidine being enriched in this pathway. Ergothioneine, a natural antioxidant and nutritional supplement synthesized from histidine, demonstrates biological functions including scavenging free radicals, mediating anti-inflammatory responses, and reducing oxidative stress [47]. Studies have demonstrated that antioxidants positively influence exercise performance and the body’s response to oxidative stress [48]. Fovet et al. found that after two hours of exercise at a consistent maximal aerobic rate, mice supplemented with ergothioneine exhibited enhanced protein synthesis and satellite cell activation, along with reduced metabolic stress, inflammatory markers, and indicators of oxidative damage [49]. Ergothioneine significantly improves aerobic performance, extends the time to post-exercise fatigue, and enhances muscle recovery. In the present study, there was a decrease in blood ergothioneine concentration in horses in AT. This reduction may be attributed to the adaptation of horses to the training intensity over time, resulting in a diminished physiological response to metabolic stress and consequently a lower secretion of ergothioneine. Further investigation is required to elucidate the specific mechanisms involved. Myostatin (β-alanyl-L-histidine), composed primarily of the amino acids L-histidine and β-alanine, is predominantly found in skeletal muscle [50]. The content of myostatin in skeletal muscle is largely influenced by factors such as age, gender, diet, muscle fiber type, and training intensity [51]. Suzuki et al. demonstrated that men with elevated myostatin levels exhibited greater strength output during the latter stages of the 30 s Wingate test [52]. Similarly, Baguet et al. found that rowers with higher myostatin levels achieved faster segment times in the second and third 500 m segments of the 2000 m race [53]. In the present study, there is a significant increase in the concentration of myostatin in the blood after training. Research has shown that myostatin may enhance exercise performance by mitigating fatigue, delaying acidosis induced by muscle contraction, and augmenting skeletal muscle force production during high-intensity exercise [54]. Additionally, purine metabolism plays a crucial role in various cellular processes, including energy storage, nucleic acid and coenzyme synthesis, translation, and signaling within the body. It serves as a metabolic pathway for both the synthesis and catabolism of purines [55]. Purines consist of three primary organic molecules: adenine derivatives (ATP, ADP, AMP, cAMP, NAD, adenosine), guanine derivatives (GTP, GDP, GMP, cGMP, guanosine), and related metabolites (hypoxanthine, xanthine, and uric acid) [56]. Research has demonstrated that the concentrations and effects of cGMP and cAMP in cells are antagonistic. For instance, elevated intracellular cAMP levels lead to the breakdown of glycogen into glucose, whereas increased cGMP levels promote the synthesis of glucose into glycogen [57]. Gaitán et al. showed that metabolomic analyses of athletes engaged in high-intensity exercise resulted in the up-regulation of lactate and adenine catabolite expression in plasma, alongside enhanced anaerobic metabolism and ATP cycling [58]. In the present study, a significant increase in cGMP concentration and a very significant decrease in 3′-adenylate concentration were observed in AT. These results are consistent with previous studies, indicating that training stimulates horses to enhance the rate of glycogen synthesis in vivo, thereby providing substantial energy for the body. Additionally, the diastole of vascular smooth muscle induces vasodilation, which increases blood flow [59], ultimately enhancing exercise performance.

Cis-aconitic acid serves as a crucial intermediate in the tricarboxylic acid cycle, which is indicative of the extent of active aerobic metabolism. This cycle represents the final metabolic pathway for the complete oxidation of the three primary energy substrates and plays a vital role in supplying energy for sustained exercise training [60]. Huang et al. demonstrated that endurance training in rats significantly enhanced both the rate of the tricarboxylic acid cycle and the organism’s antioxidant activity, resulting in increased levels of intermediates such as pyruvate, malate, and aconitate [61]. In the present study, there was a significant increase in the concentration of cis-aconitic acid in the blood of horses after training. This observation suggests that the training process stimulates the acceleration of the tricarboxylic acid cycle, leading to the release of more intermediates that provide energy to the body, thereby enhancing the endurance and athletic performance of horses.

This study selected 2-year-old trot-type Yili horses as experimental subjects. Although horses at this age stage are in a period of rapid development of athletic capacity with a relatively stable physiological status, age-related factors may still present the following potential risks: 2-year-old horses are in a critical period of skeletal muscle development and metabolic system maturation, where gene expression networks may exhibit dynamic regulatory characteristics due to changes in growth hormone and sex hormone levels. The observed differences in specific gene expression may be partially attributed to age-dependent regulation rather than solely reflecting training effects. Under identical training protocols, individual variations in gene expression and metabolic adaptation may be amplified by age factors. Therefore, the conclusions of this study may be specific to this particular developmental stage and cannot be generalized to horses of other age groups. Future research could further incorporate horses from different age groups to comprehensively evaluate the impact of age on relevant gene expression and metabolic regulation.

## 5. Conclusions

In summary, through the integrated analysis of transcriptomics and metabolomics, seven genes—*CCL5*, *CCR3*, *FOS*, *CD3E*, *HSD17B1*, *C1QTNF12*, and *GATA1*—were identified. These genes are primarily associated with the athletic performance of trot-type Yili horses, highlighting their importance in the influence of training on equine exercise performance. Further investigation into their functions will aid in enhancing the athletic performance of trot-type Yili horses. Subsequent studies are required to assess their applicability to other horse breeds.

## Figures and Tables

**Table 1:** Specialized trot conditioning training program.

Training Stage	Time	Training Content
Before training	Week 1	Completion of horse affinity training.
	Week 2	Single rein conditioning circle training 20 min/day: 10 min each on the right and left inner arms.
	Week 3	Single rein conditioning circle training 20 min/day: after 10 min each in the right and left inner arms, saddle preparation training.
	Week 4	Double rein conditioning circle training 30 min/day: 15 min each in the right and left inner arms.
Mid-training	Week 5	Double rein conditioning circle training 40 min/day: 20 min each in the right and left inner arms.
	Week 6	Warm-up training for the conditioning circle, 5 min each on the right and the left side of the body. Walk grass training, two laps each of the forward and reverse lines.
	Week 7	Warm-up training for the conditioning circle, 5 min each on the right and the left side of the body. Grass route training, 15 min for each route.
	Week 8	Warm-up training for the conditioning circle, 5 min each on the right and the left side of the body. One lap each of positive and negative lines for sand slow backpedal training for sand lines.
After training	Week 9	Warm-up training for the conditioning circle, 5 min each on the right and the left side of the body. Riding training walk for 2000 m.
	Week 10	Warm-up training for the conditioning circle, 5 min each on the right and the left side of the body. Riding training walk for 2000 m, trot for 1000 m.
	Week 11	Warm-up training for the conditioning circle, 5 min each on the right and the left side of the body. Riding training (walk + trot) for 2000 m.
	Week 12	Warm-up training for the conditioning circle, 5 min each on the right and the left side of the body. Riding training (walk + trot) for 2000 m.

**Table 2:** Race results of horses at different stages of training.

Items	Before-Training	Mid-Training	After-Training
Race time (s)	336.62 ± 36.06 ^Aa^	316.01 ± 26.35 ^ABb^	303.48 ± 25.51 ^Bb^

Note: Different lowercase superscript letters indicate significant differences (*p* < 0.05), while different uppercase superscript letters denote highly significant differences (*p* < 0.01).

**Table 3:** Sequencing quality and number of reads.

Sample	Raw Reads	Clean Reads	Error Rate	Q20%	Q30%	GC Content%	Mapped Reads
BT88	46,659,636	72,290	0.03	96.49	91.77	53.13	16,742 (23.16%)
BT104	47,835,518	45,103,352	0.03	97.10	92.16	52.60	36,975,171 (81.98%)
BT105	47,867,242	194,802	0.03	96.78	92.11	57.96	163,550 (83.96%)
BT72	49,818,952	1,360,372	0.03	97.23	92.74	56.90	1,028,107 (75.58%)
AT88	31,903,812	31,808,328	0.03	96.84	91.77	51.34	21,458,031 (67.46%)
AT104	46,635,024	156,744	0.03	96.78	92.15	58.40	96,856 (61.79%)
AT105	45,383,768	612,552	0.03	96.66	92.10	56.32	506,631 (82.71%)
AT72	43,102,918	40,677,736	0.03	97.06	92.03	52.23	38,359,904 (94.3%)

BT, before-training; AT, after-training.

**Table 4:** Important differential genes between after training and before training.

Genes	log_2_FC	*p*-Value	Up/Down
CCL5	1.513	0.027	Up
FOS	3.193	0.001	Up
CD3E	1.819	0.012	Up
HSD17B1	3.635	0.046	Up
CCR3	−2.316	0.022	Down
C1QTNF12	−3.185	0.011	Down
GATA1	−3.098	0.016	Down
TMPRSS6	2.310	0.049	Up
CLEC1A	−3.624	0.025	Down
PRF1	2.711	0.002	Up
ND5	−1.657	0.029	Down

**Table 5:** Important differential metabolites between after training and before training.

Metabolites	VIP	FC	log_2_FC	*p*-Value	Up/Down
N-Acetyl-L-leucine	1.402	1.585	0.664	0.023	Up
Dehydroepiandrosterone (DHEA)	1.308	1.780	0.832	0.041	Up
cis-Aconitic acid	1.342	3.437	1.781	0.033	Up
Pentadecanoic Acid	1.278	0.416	−1.266	0.014	Down
Indoxyl-β-D-glucuronide	1.594	20.878	4.384	0.029	Up
Palmitoyl sphingomyelin	1.486	1.702	0.768	0.041	Up
3′-Adenosine monophosphate (3′-AMP)	1.485	0.082	−3.612	0.001	Down
Proline-hydroxyproline	1.691	1.662	0.733	0.005	Up
Asparagine	1.116	0.659	−0.601	0.014	Down
Acetaminophen glucuronide	1.310	2.664	1.413	0.001	Up
Carnosine	1.647	1.874	0.906	0.021	Up
Pantothenic acid	1.177	1.560	0.642	0.024	Up
Ergothioneine	1.665	0.476	−1.070	0.022	Down
Androsterone	1.618	0.177	−2.498	0.016	Down
Nicotinuric Acid	1.342	0.439	−1.188	0.023	Down
Fmoc-L-Isoleucine	1.504	1.634	0.708	0.027	Up
Prostaglandin J2	1.235	0.382	−1.388	0.013	Down
cGMP	1.141	1.507	0.592	0.028	Up
1-Methylhistidine	1.709	2.223	1.152	0.008	Up
Adenosine 5′-monophosphate	1.456	0.116	−3.109	0.001	Down

## Data Availability

The original contributions presented in the study are included in the article, further inquiries can be directed to the corresponding author.
